# Analysis of age-dependent gene-expression in human tissues for studying diabetes comorbidities

**DOI:** 10.1038/s41598-023-37550-x

**Published:** 2023-06-26

**Authors:** Pietro Hiram Guzzi, Francesca Cortese, Gaia Chiara Mannino, Elisabetta Pedace, Elena Succurro, Francesco Andreozzi, Pierangelo Veltri

**Affiliations:** 1 Department of Surgical and Medical Sciences, Magna Graecia University, 88100 Catanzaro, Italy; 2 Internal Medicine Unit, R. Dulbecco Hospital, 88100 Catanzaro, Italy; 3 Internal Medicine Unit, ASP Catanzaro, Soverato Hospital, Soverato, Italy; 4 DIMES, University of Calabria, Rende, Italy

**Keywords:** Computational biology and bioinformatics, Molecular medicine

## Abstract

The study of the relationship between type 2 diabetes mellitus (T2DM) disease and other pathologies (comorbidities), together with patient age variation, poses a challenge for medical research. There is evidence that patients affected by T2DM are more likely to develop comorbidities as they grow older. Variation of gene expression can be correlated to changes in T2DM comorbidities insurgence and progression. Understanding gene expression changes requires the analysis of large heterogeneous data at different scales as well as the integration of different data sources into network medicine models. Hence, we designed a framework to shed light on uncertainties related to age effects and comorbidity by integrating existing data sources with novel algorithms. The framework is based on integrating and analysing existing data sources under the hypothesis that changes in the basal expression of genes may be responsible for the higher prevalence of comorbidities in older patients. Using the proposed framework, we selected genes related to comorbidities from existing databases, and then analysed their expression with age at the tissues level. We found a set of genes that changes significantly in certain specific tissues over time. We also reconstructed the associated protein interaction networks and the related pathways for each tissue. Using this mechanistic framework, we detected interesting pathways related to T2DM whose genes change their expression with age. We also found many pathways related to insulin regulation and brain activities, which can be used to develop specific therapies. To the best of our knowledge, this is the first study that analyses such genes at the tissue level together with age variations.

## Introduction

The co-occurrence of diseases, or comorbidity, may have both a genetic and environmental cause^1^. Comorbidities, especially with chronic diseases, cause a rapid decline in the quality of life and longevity. Moreover, they contribute to an increased demand for hospital beds and higher costs for the health care system overall^2^. Consequently, there is the need to shed light on the insurgence and progression of comorbidities by elucidating the time of their onset and their genetic relations^3–5^. Moreover, comorbidities can vary with age, sex, and external factors such as environmental issues related to living areas^6–9^.

In this work, we focus on diabetes mellitus disease and its comorbidities. Diabetes mellitus, with an estimated number of 415 million adults affected, is one of the most widespread chronic diseases^10^. There are three types of diabetes: type 1 Mellitus (T1DM), type 2 Mellitus (T2DM), and gestational diabetes. Here we consider comorbidities in T2DM, considered the most common category of diabetes mellitus^11–15^. T2DM is a complex metabolic disorder characterised by a progressive loss of b-cell insulin secretion, causing hyperglycemia against a background of insulin resistance. It often presents at least one comorbidity in patients^16–19^. T2DM, prevalent in adults over 65 years^7,10,20,21^, considerably impacts public health causing high mortality, disability and hospitalisation^22^. The underlying pathophysiology of the disease is exacerbated by the ageing process, which affects metabolic regulation and accelerates the progression of many comorbidities^23–26^. For instance, in people aged 65–79, diabetes mellitus is associated with a high risk of cardiovascular, microvascular, and other complications^27–29^.

**Figure 1:**
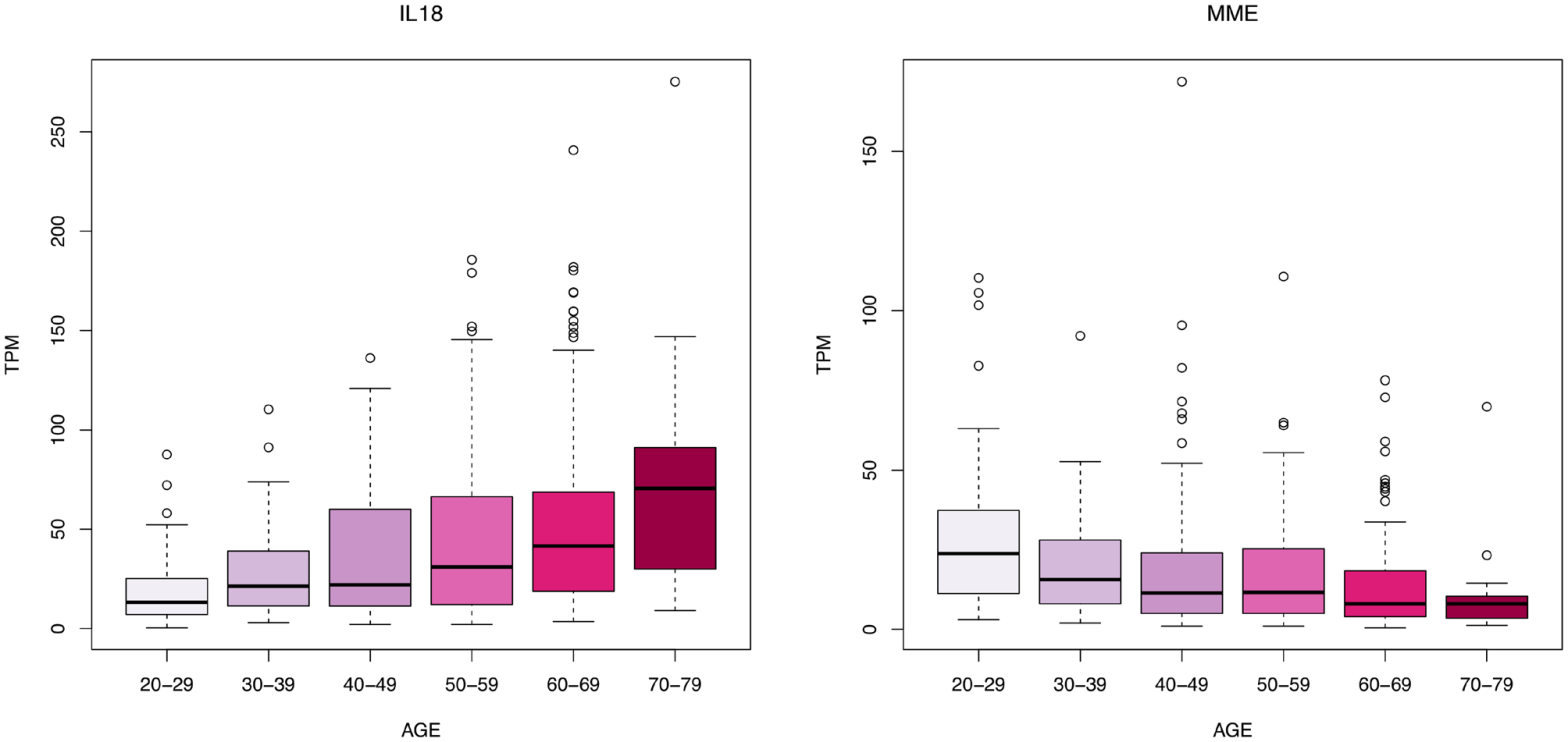
Boxplots illustrating two gene expressions patterns: an increasing gene expression (on the left representing the IL18 gene) and a decreasing one on the right (the MME gene).

**Figure 2:**
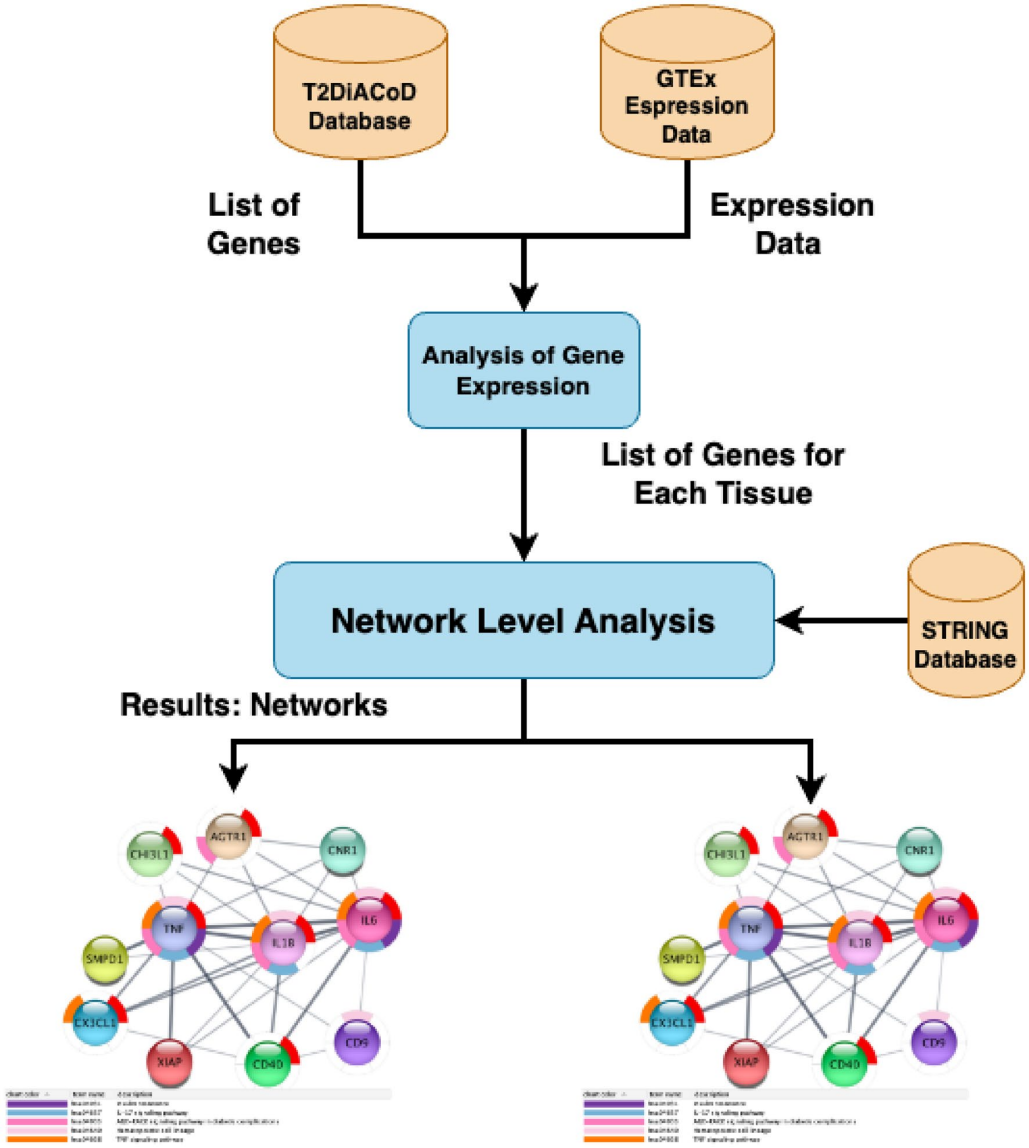
Figure depicts the workflow of the experiment performed in the proposed framework. First, we select the list of genes related to comorbidities of T2DM on the T2DiACoD database. Then, we retrieve expression data on GTEx data portal for each previously selected gene. Metadata regarding the age and sex of patients are also considered. We then find those genes that exhibit an increase (or decrease) of mean expression changes with age. Protein interaction networks corresponding to the genes with significant changes are gathered from the STRING database and then analysed.

**Figure 3:**
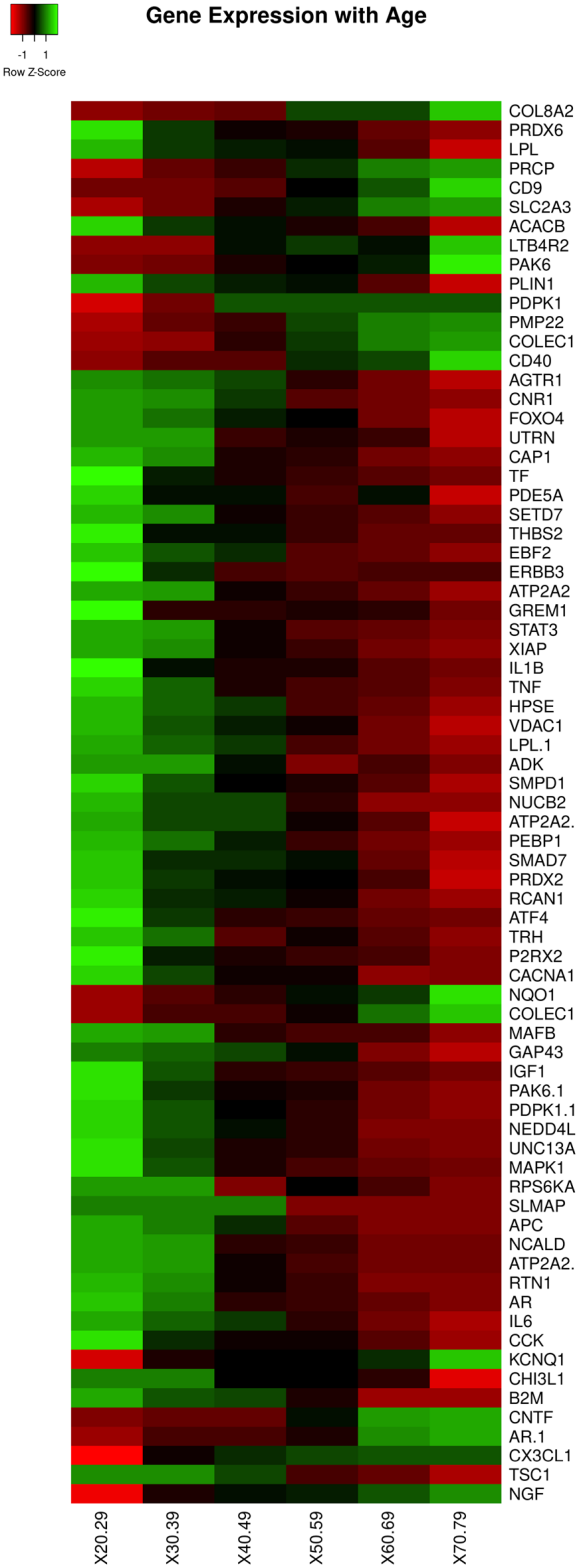
Figure depicts the average value of the expression of genes. Rows represent genes with significant changes (by means of a Kruskal Wallis test). Columns represent age groups: 20–29, 30–39. 40–49, 50–59, 60–69, 70–79 years. Lower values are represented in red while green means higher values.

We study T2DM comorbidities by leveraging existing data and models at the system level^30,31^ and integrating information by means of network medicine models. Network medicine (together with data science) can be used as a practical framework for studying disease comorbidities and progression^1,32–38^. It is based on the analysis of data from graph theory model integrating different data sources. In this work, we propose a mechanistic framework to study molecular causes related to comorbidities at a tissue level related to age. We hypothesise that differences in age may be explained by differences at the molecular level of genes whose basal expression is modified with age. For instance, Fig. 1 depicts the behaviour of an increasing and a decreasing gene expression with respect to different human age groups. To this end, we consider genes for which there is evidence of correlation with comorbidities in T2DM disease. By using T2DiACoD database^39^ we study the genes correlated to T2DM comorbidities. Indeed, the T2DiACoD database contains genes that provide evidence of correlation with comorbidities in T2DM. Also, we consider tissues associated with genes and their variations with age. The proposed framework allows us to: (i) identify genes presenting significant changes with ageing and (ii) define networks at the tissue level in order to study significant gene changes at the network level. The workflow implemented by the proposed framework is reported in Fig. 2. We start from gene expression data stored in the GTEx database^40^ and comorbidities of TD2M stored in the T2DiACO database. Focusing on T2DM, we obtained results from 54 different tissues organised into six age groups 20-29, 30-39, 40-49, 50-59, 60-69, 70-79 years (see Fig. 2). We obtained different samples for each tissue, and then calculated the median value of the expression in each age interval. We then filter genes presenting a significant increase or decrease with age as reported in Fig. 3.

**Table 1:** Table reports the number of genes which present a modification (number of genes increasing and decreasing) for different age groups in different tissues.

Tissue	No increasing	No decreasing
Adipose subcutaneous	9	4
Adipose visceral	51	17
Adrenal gland	7	4
Artery aorta 2022	5	5
Artery tibial 2022	0	0
Brain anterior	1	11
Brain caudate	5	2
Brain cerebellar hemisphere	3	24
Brain cerebellum	3	20
Brain cortex	13	12
Brain frontal cortex	1	6
Brain hippocampus	4	20
Brain hypotalamus	1	14
Brain	2	4
Brain putamen basal ganglia	3	3
Brain spinal cord cervical	0	2
Brain substantia nigra	1	17
Breast mammary 202	1	1
Colon sigmoid	0	1
Colon transverse	2	0
Esophagus muscolaris	1	0
Heart atrial appendage	3	0
Heart left ventricle	1	0
Kidney cortex	0	2
Liver	2	8
Lung	2	5
Minor salivary gland	9	2
Muscle skeletal	10	0
Nerve tibial	2	1
Ovary	1	1
Pancreas	12	0
Pituitary	3	0
Prostate	4	1
Spleen	1	1
Testis	1	0
Thyroid	0	0
Uterus	6	13
Vagina	0	1
Blood	1	2
Total	171	204

Our results show that there is a considerable number of genes whose expression changes with age and thus related to the risk of presenting comorbidities with T2DM. Finally, the proposed framework uses the STRING^41^ database for deriving the protein interaction networks induced by the genes obtained in the above steps (i.e., extracting the protein interaction networks corresponding to the identified genes in T2DiACO database). With a multiscale approach, the obtained networks can thus be studied by means of tissues, age and gender as factors^42^.

Using the here proposed framework, we obtained results suggesting that ageing may augment the risk of T2DM comorbidities. This indicates the need for further research on the mechanisms of the age-associated increase in the prevalence of T2DM, which can also be used for novel therapeutic strategies^43^.

## Results

Figure 2 reports the workflow of the framework used to analyze genes related to T2DM disease. The framework identifies genes whose expression changes with age. Then selected genes are used to define protein interaction networks that can be associated with tissues. Networks (see Finally, see bottom part of Figure 2) are then studied and analyzed to identify those genes that exhibit an increase (or decrease) of mean expression changes with age.

**Figure 4:**
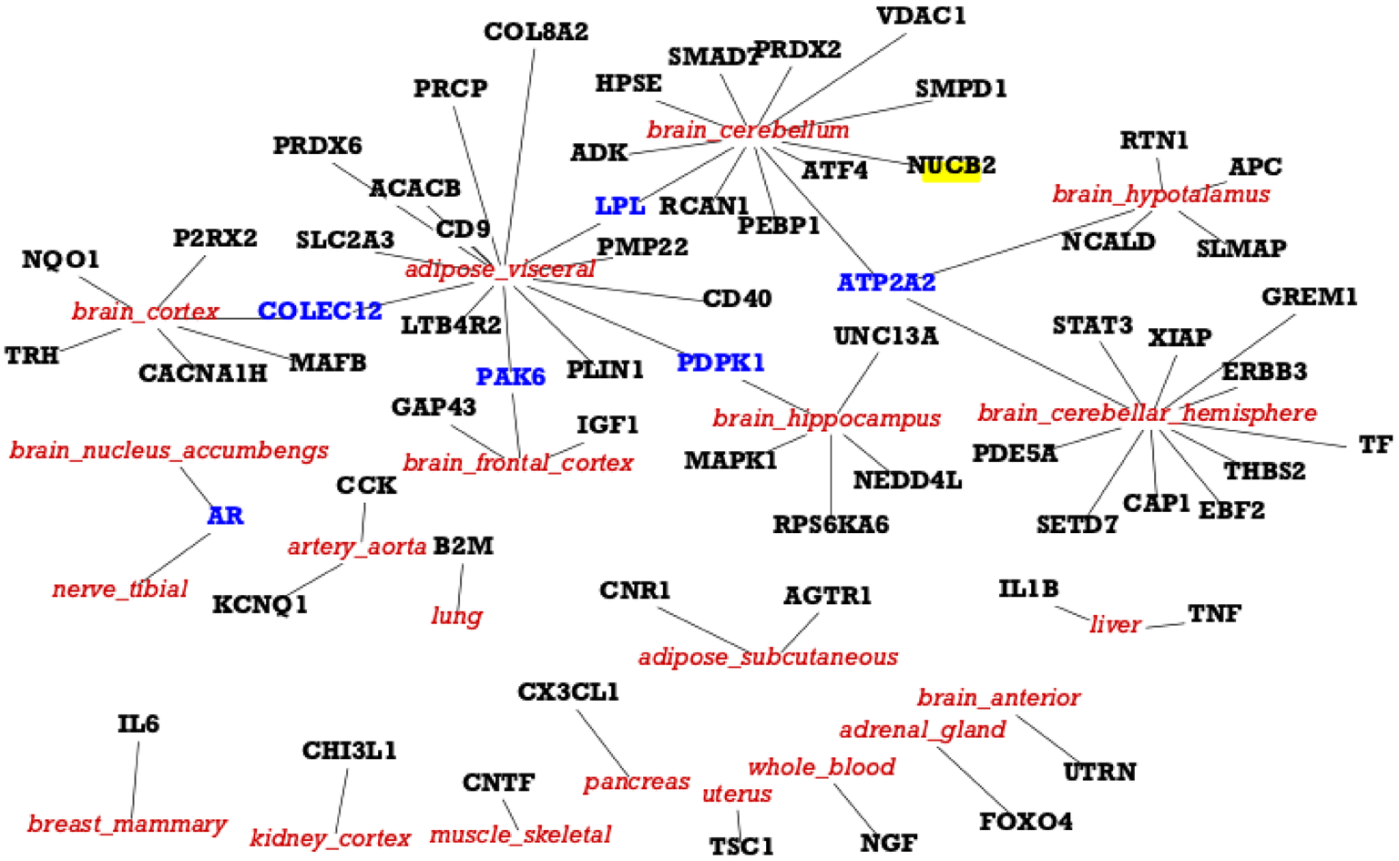
Figure reports the genes which present a significant modification over time in different tissues (*p* value <0.05, Kruskal Wallis Test). The figure represents the association between genes (in bold) and tissues (in red italics). Blue labels evidence genes we found changed in more tissues.

**Figure 5:**
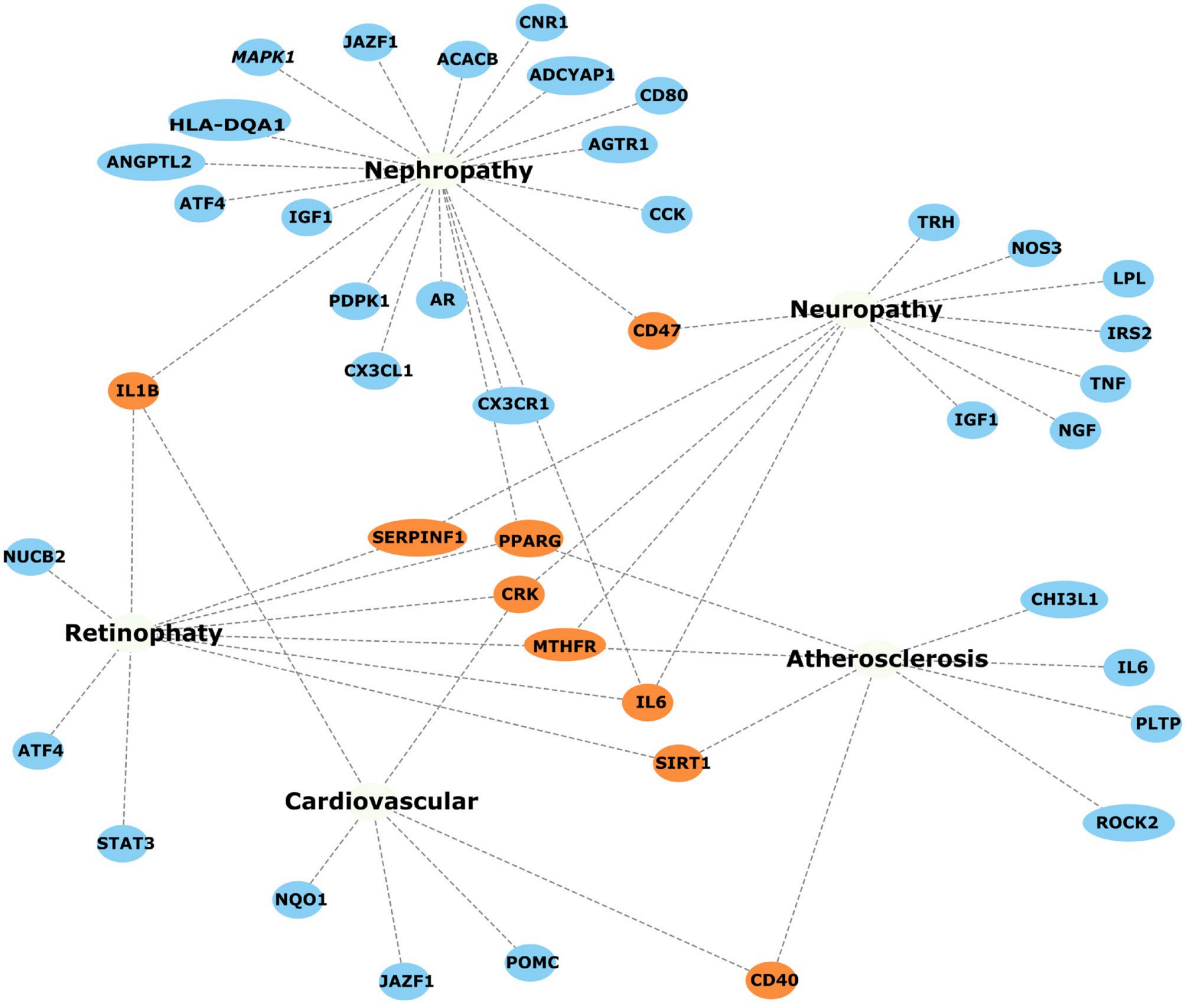
Figure highlights the relations among the studied genes and the comorbidities, as reported in the T2DiACoD database. Blue Nodes are related to a single disease, while orange nodes are shared between two or more diseases.

Starting from genes in the T2DiACoD database, we extract their associated expression values from the GTEx database, where each expression is related to tissues and human-age interval (e.g., gene expression for liver in the 30-40 years age range). For each tissue we select only *increasing* or *decreasing* genes. We found 171 increasing genes (some of which are present in more than one tissue), and 204 decreasing genes. We then selected only genes with a significant change measured by means of Kruskal–Wallis test^44^. We also performed the functional (Gene Ontology) and pathway (KEGG database)^45^ enrichment analysis^46^. Table 1 reports the number of genes with changes for each tissue, while Fig. 4 reports the associations among genes and tissues. *We also evidenced the relations among the genes and the associated comorbidities in Fig.*5, *where relations are extracted from the T2DiACoD database.* Figure 3 depicts the behaviour only of significantly increasing and decreasing genes in the heatmap.

**Figure 6:**
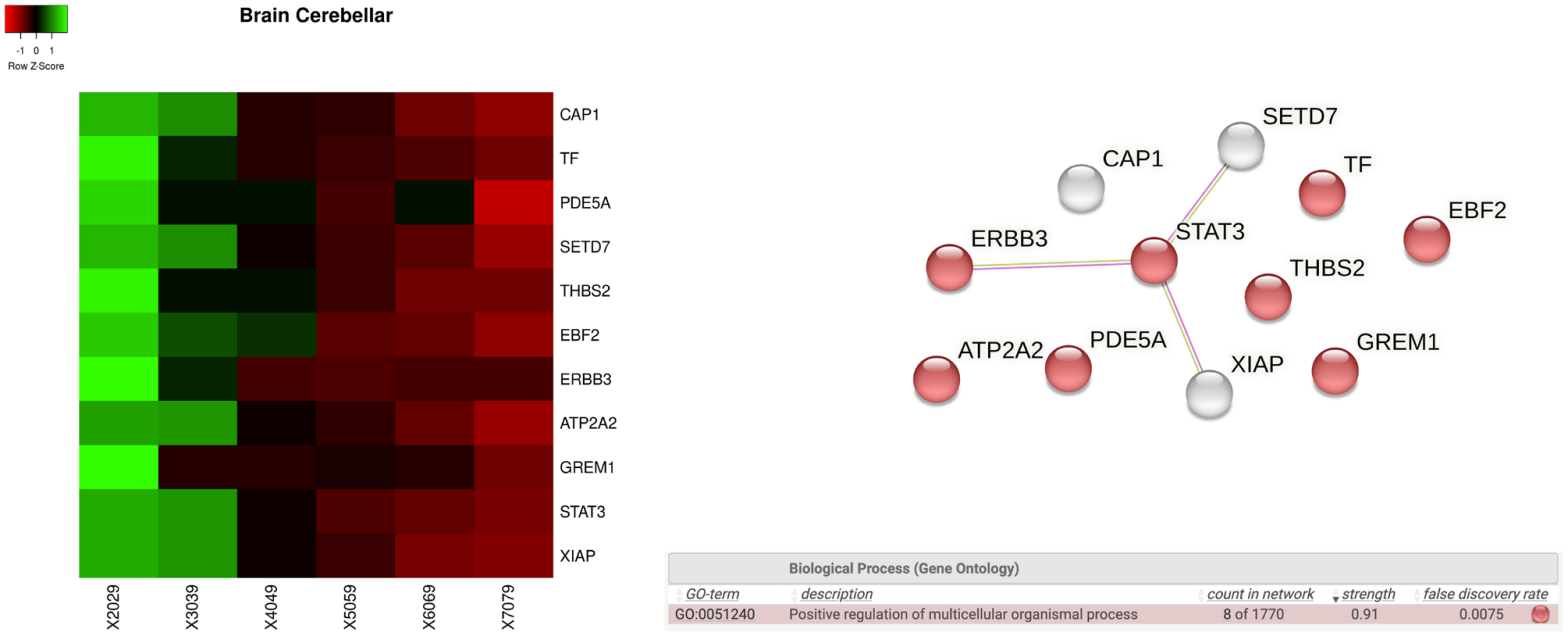
Figure reports the heatmap of the genes with significant changes in *Brain Cerebellar tissue* with age. Changes have been measured by using a Kruskal–Wallis test. Rows of the heatmap represent genes with significant changes. Columns represent age groups: 20–29, 30–39. 40–49, 50–59, 60–69, 70–79 years. Lower values are represented in red while green means higher values. Figure depicts the protein interaction network extracted from the STRING database. Figure bottom part reports functional enrichment analysis (biological process from Gene Ontology portal).

**Figure 7:**
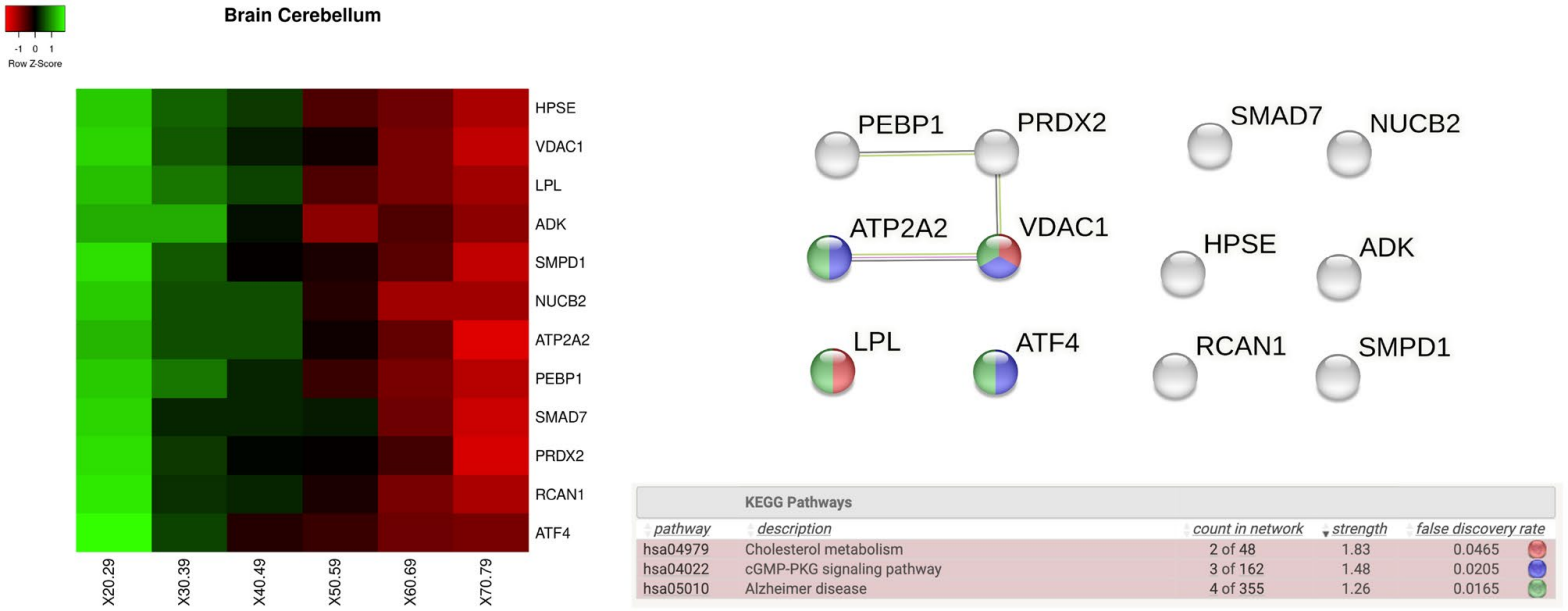
Figure depicts the heatmap of the genes with significant changes in *Brain Cerebellum* tissue with age. Changes have been measured by using a Kruskal–Wallis test. Rows of the heatmap represent genes with significant changes. Columns represent age groups: 20–29, 30–39. 40–49, 50–59, 60–69, 70–79 years. Lower values are represented in red while green means higher values. Figure depicts the related protein interaction network extracted from the STRING database while the bottom part contains the functional enrichment analysis (biological process from Gene Ontology portal).

We analyse gene expressions in different tissues, and for each tissue, we investigate the behaviour of significantly increasing and decreasing genes as heatmap by means of a Kruskal Wallis test. We also focus on the protein interaction networks gathered from the STRING (when available) annotated with the main pathways found. For each tissue, we measured the functional enrichment by using the STRING database, where a functional enrichment is considered significant when the false discovery rate (FDR) is less than 0.01. For *brain cerebellum* and *brain cerebellar haemisphere* tissues we found the following genes: *HPSE, VDAC1 LPL, ADK, SMPD1, NUCB2, ATP2A2, PEBP1, SMAD7, PRDX2, RCAN1, and ATF4*. Such genes are reported as changes of expression as heatmaps in Figs. 6 and 7 with respect to age intervals. Rows represent genes with significant changes while columns represent age groups: 20–29, 30–39. 40–49, 50–59, 60–69, 70–79 years old (i.e. 20–29 notation indicates an age interval among 20 and 29 years old). Both Figures report the proteins interaction where proteins are related to the found genes.

**Figure 8:**
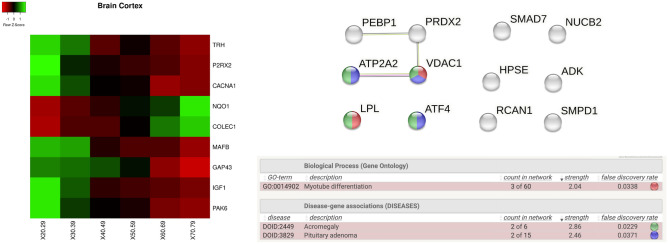
Figure depicts the heatmap of the genes with significant changes in *Brain Cortex tissue* with age. Changes have been measured by using a Kruskal–Wallis test. Rows of the heatmap represent genes with significant changes. Columns represent age groups: 20–29, 30–39. 40–49, 50–59, 60–69, 70–79 years. Lower values are represented in red while green means higher values. Interaction network for proteins extracted from the STRING database is reported in the middle, while the bottom part contains the functional enrichment analysis (biological process from Gene Ontology portal).

In *brain cortex* tissue we found significant changes for the following genes, whose behaviour is reported as heatmap in Fig. 8: *TRH, P2RX2, CACNA1H, NQO1, COLEC12, MAFB*. As the above tissues, Fig. 8 reports the protein interactions network built by gathering data from STRING database.

**Figure 9:**
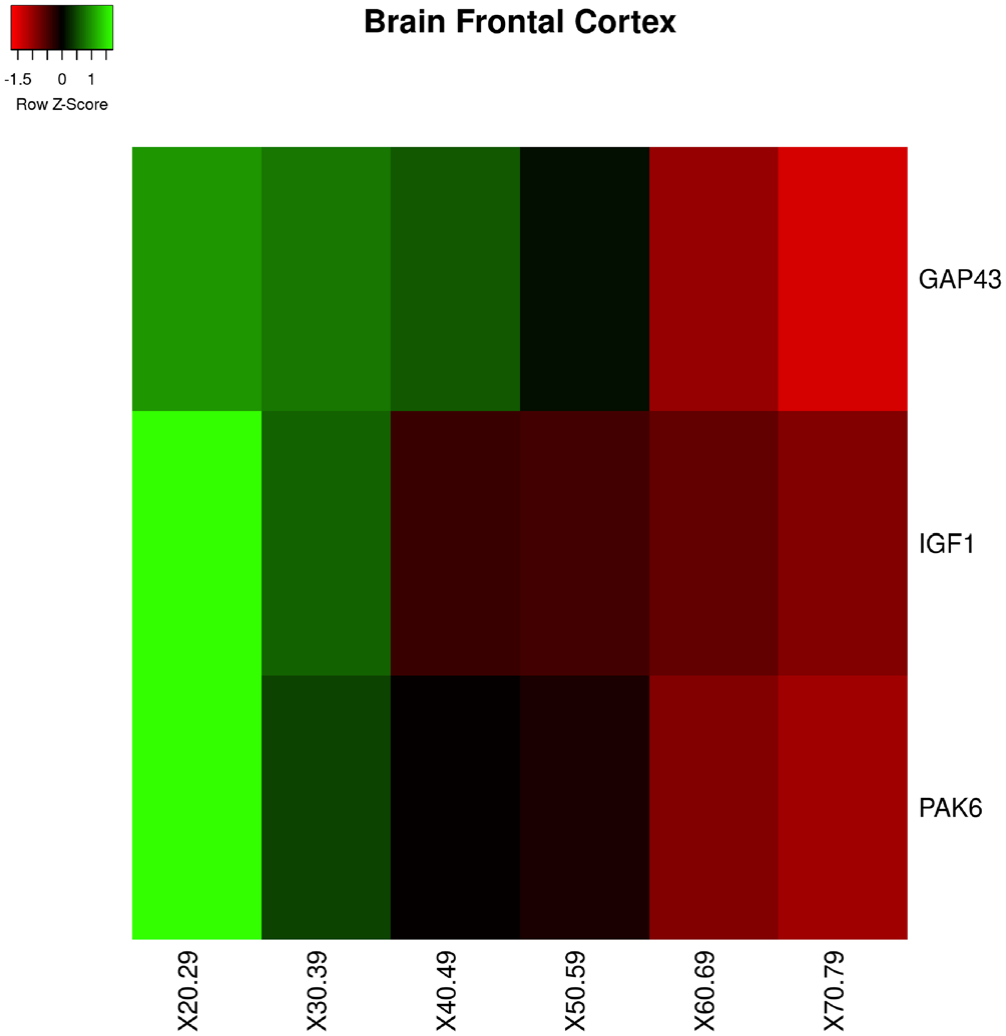
Figure depicts the heatmap of the genes with significant changes in *Brain Frontal tissue* with age. Changes have been measured by using a Kruskal–Wallis test. Rows of the heatmap represent genes with significant changes. Columns represent age groups: 20–29, 30–39. 40–49, 50–59, 60–69, 70–79 years. Lower values are represented in red while green means higher values. The interaction network gathered from STRING databases is the one related to such genes. The bottom part contains the functional enrichment analysis (biological process from Gene Ontology portal).

In *brain frontal cortex* tissue we found significant changes for the following genes: *GAP43, IGF1, PAK6*. Fig. 9 reports the behaviour of such genes as heatmap and the protein interactions network built by **querying** STRING.

**Figure 10:**
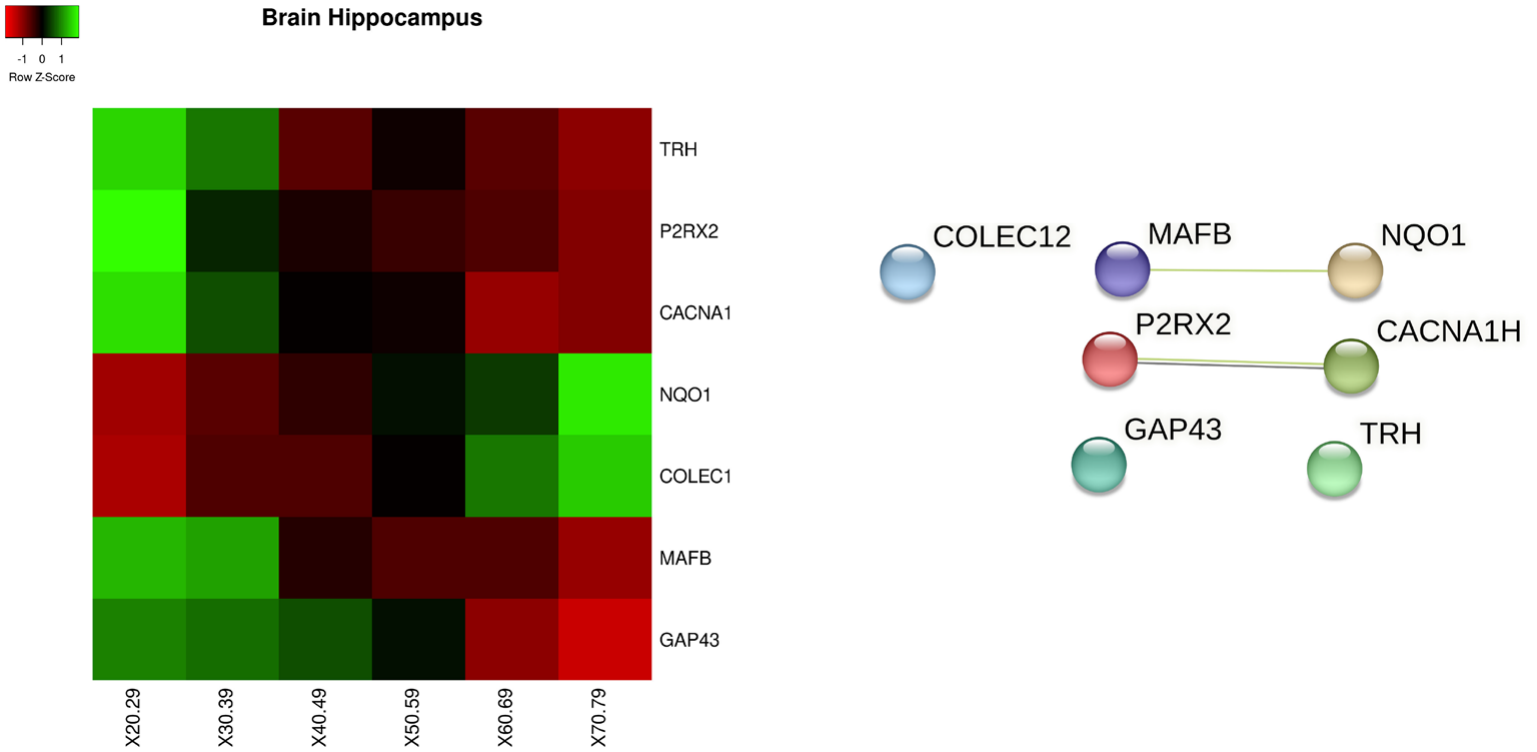
Figure depicts on the left the heatmap of the genes with significant changes in *Brain hippocampus* tissue with age. Changes have been measured by using a Kruskal–Wallis test. Rows of the heatmap represent genes with significant changes. Columns represent age groups: 20–29, 30–39. 40–49, 50–59, 60–69, 70–79 years. Lower values are represented in red while green means higher values. The central area of the figure depicts the related protein interaction network extracted from the STRING database while the bottom part contains the functional enrichment analysis (biological process from Gene Ontology portal).

For the *hippocampus* tissue we detected *PDPK1, NEDD4L, UNC13A, MAPK1, and RPS6KA6* genes, whose values are reported as heatmap in Fig. 10.

**Figure 11:**
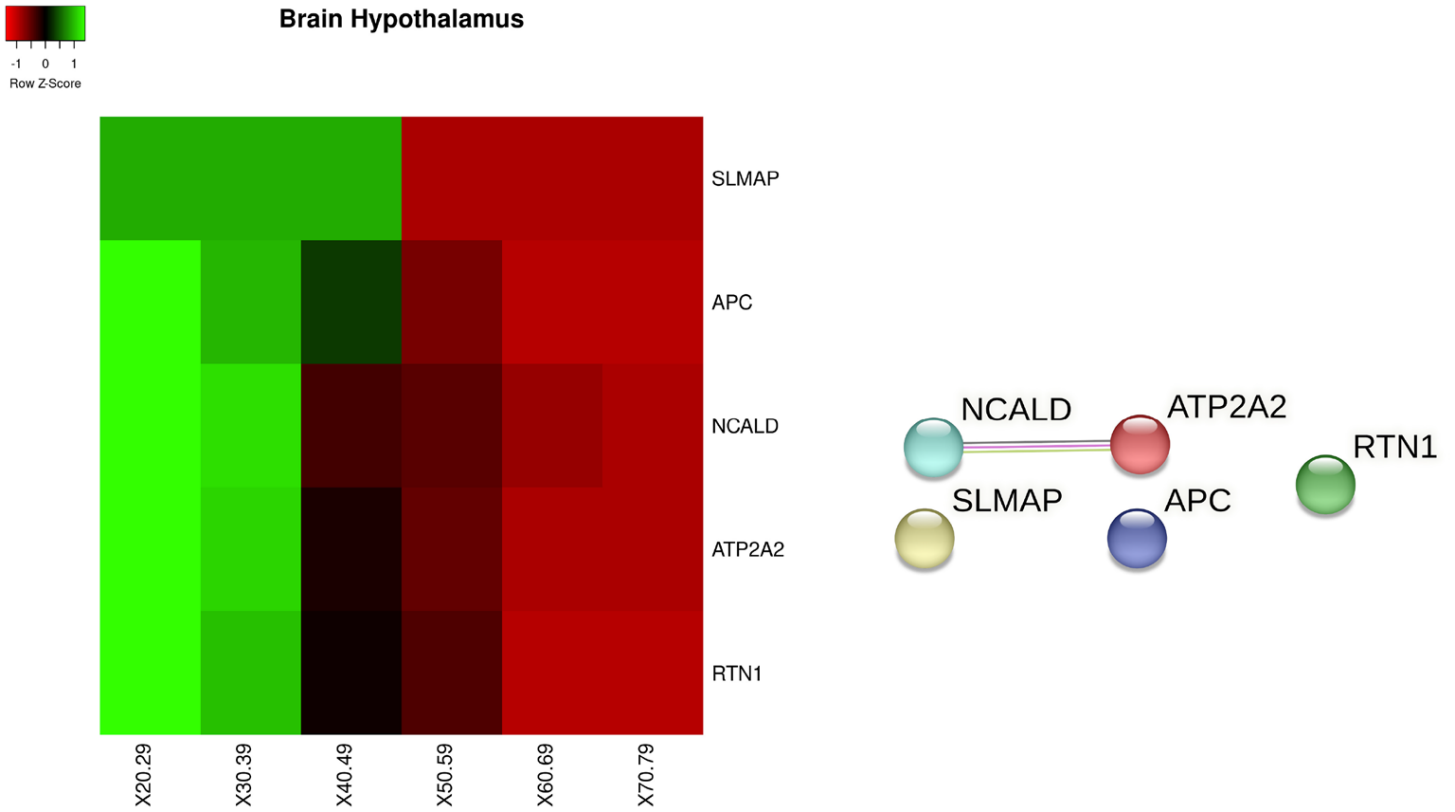
Figure depicts on the left the heatmap of the genes with significant changes in *Brain hypothalamus tissue* with age. Changes have been measured by using a Kruskal–Wallis test. Rows of the heatmap represent genes with significant changes. Columns represent age groups: 20–29, 30–39. 40–49, 50–59, 60–69, 70–79 years. Lower values are represented in red while green means higher values. The central area of the figure depicts the related protein interaction network extracted from the STRING database while the bottom part contains the functional enrichment analysis (biological process from Gene Ontology portal).

**Figure 12:**
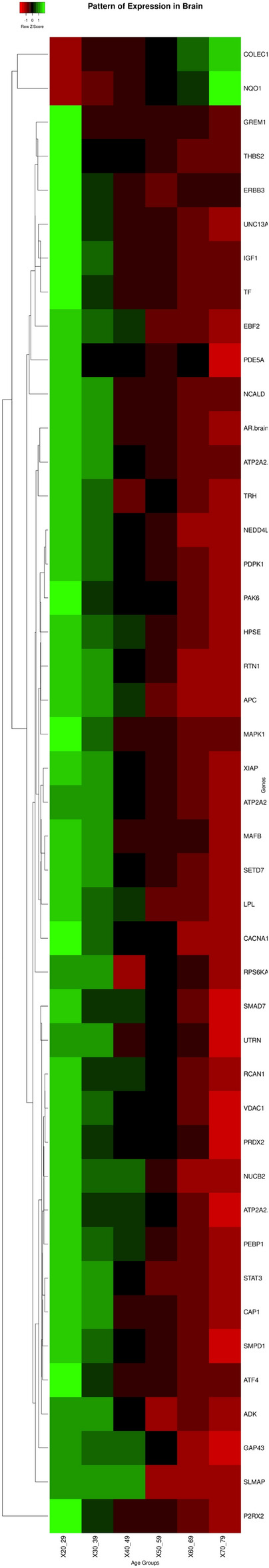
Heatmap showing the behaviour of all the significant changed genes in all brain tissues divided by age interval (i.e. 20–29,...).

In the *hypothalamus*, we found *SLMAP, APC, NCALD, ATP2A2, and RTN1* genes as reported in Fig. 11. Figure 12 reports the set of identified genes for all the aforementioned brain tissues.

**Figure 13:**
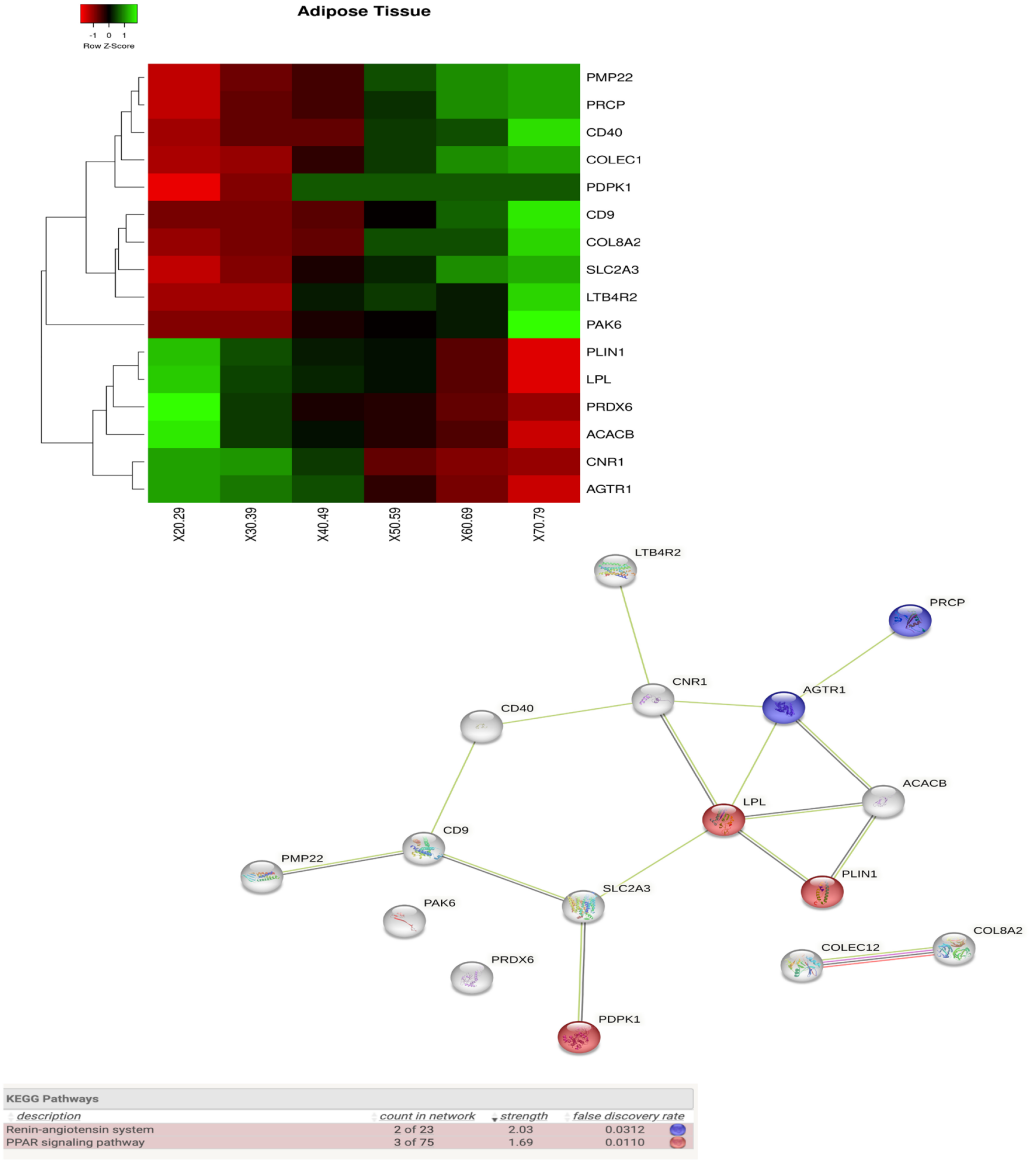
Figure depicts on the left the heatmap of the genes with significant changes in *Adipose * tissue with age. Changes have been measured by using a Kruskal–Wallis test. Rows of the heatmap represent genes with significant changes. Columns represent age groups: 20–29, 30–39. 40–49, 50–59, 60–69, 70–79 years. Lower values are represented in red while green means higher values. The central area of the figure depicts the related protein interaction network extracted from the STRING database while the bottom part contains the functional enrichment analysis (biological process from Gene Ontology portal).

Furthermore, the results are related to different tissues. We found a relatively high number of modulated genes in *adipose* tissue, in particular the following: *COL8A2, PRDX6, LPL, PRCP, CD9, SLC2A3, ACACB, LTB4R2, PAK6, PLIN1, PDPK1, PMP22, COLEC12, CD40, AGTR1, and CNR1*. The heatmap in Fig. 13 reports the genes found in the tissue grouped by age and the protein networks obtained by querying STRING.

**Figure 14:**
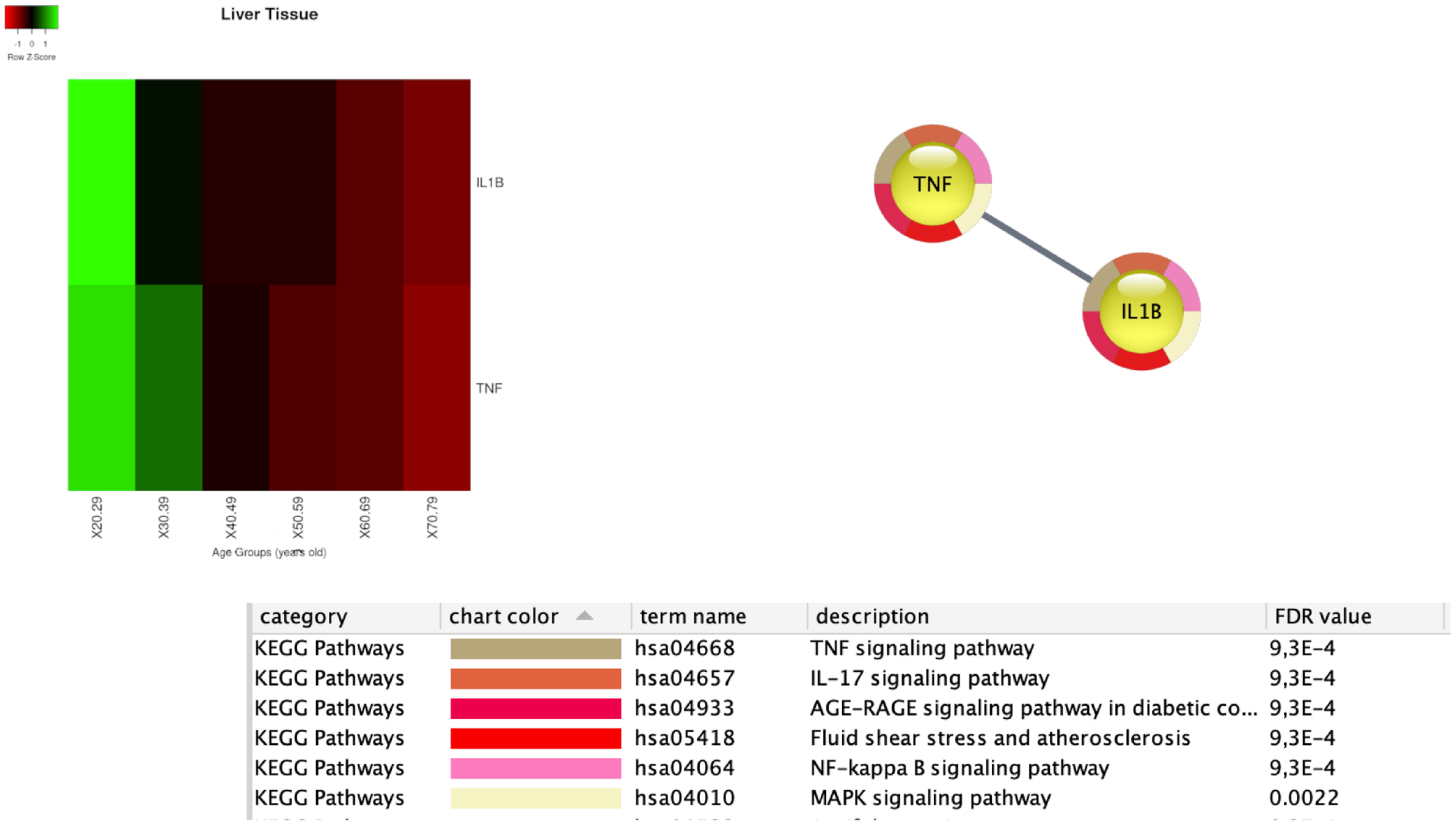
The top left corner of the figure depicts the average value of the gene expressions. Rows represent genes with significant changes (by means of Kruskal–Wallis test). Columns represent age groups: 20–29, 30–39. 40–49, 50–59, 60–69, 70–79 years. Lower values are represented in red while green means higher values. The central area depicts the related protein interaction network extracted from the STRING database and the pathway analysis, at the bottom.

For *liver* tissue, we found expression changes for IL1B and TNF genes, as reported in Fig. 14.

**Figure 15:**
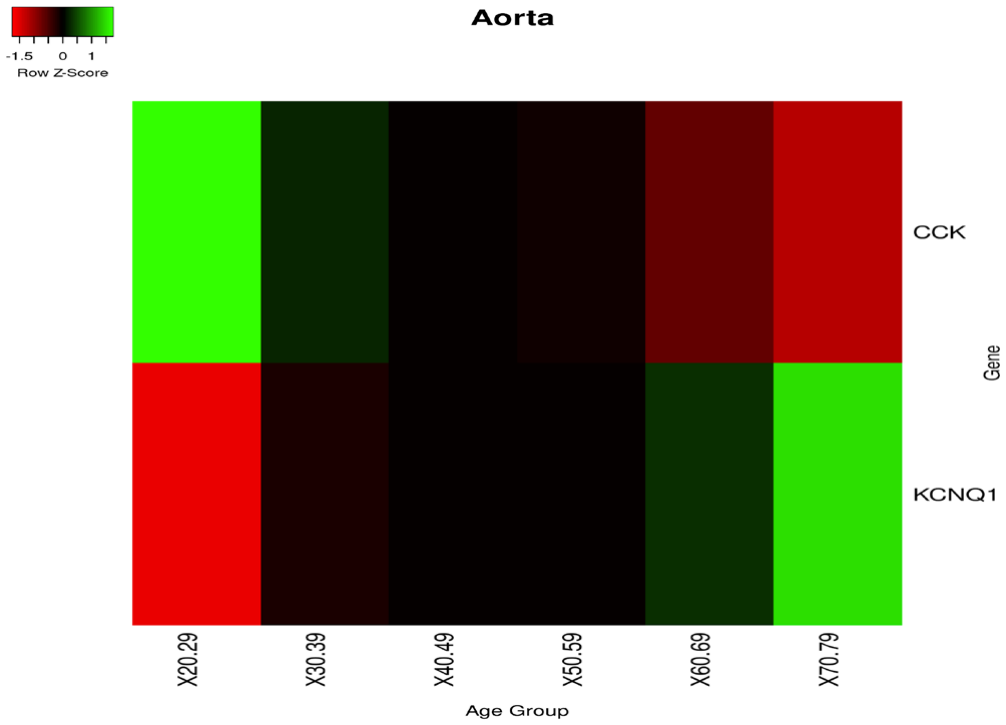
Gene expression in **aorta** tissue. We found CCK that decreases with age while KCNQ1 increases. Significant changes found by means of a Kruskal–Wallis Test, *p*<0.05.

In the *aorta* tissue, we found the modulation of CCK and KCNQ1 genes (see Fig. 15).

**Figure 16:**
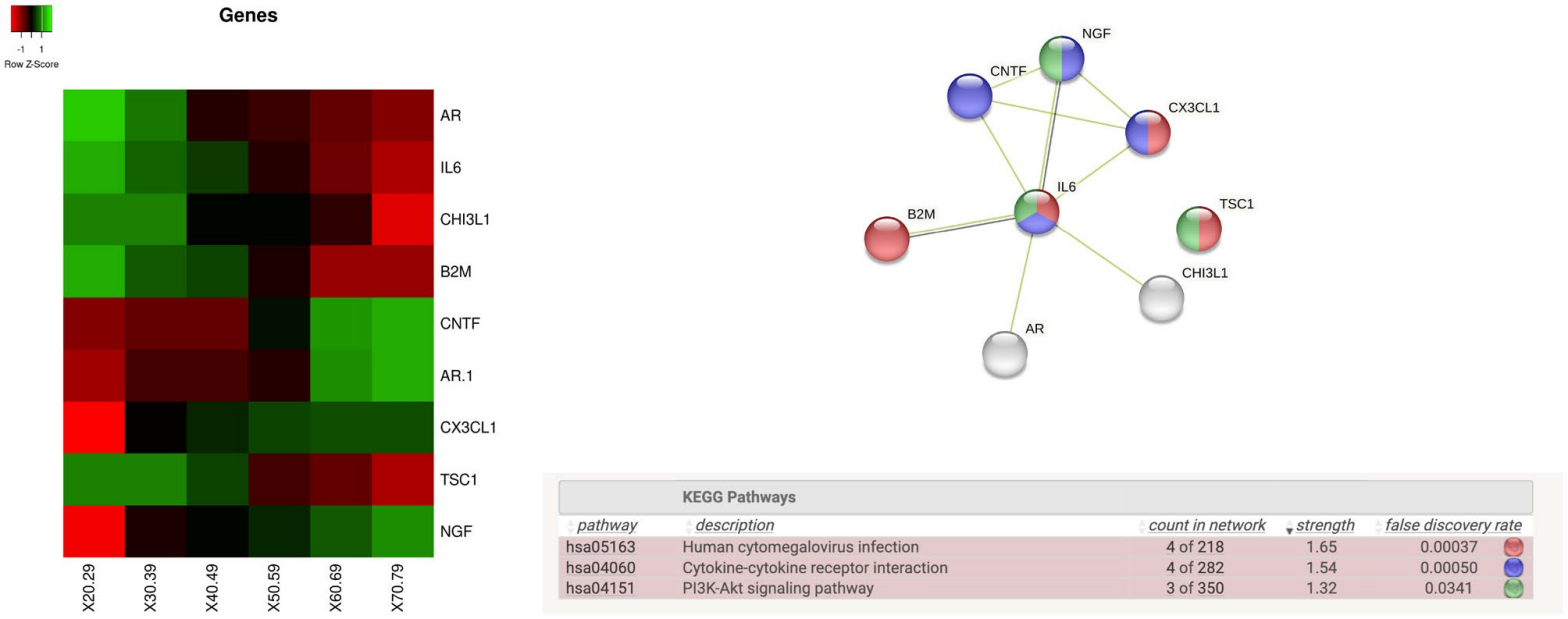
The top left corner of the figure depicts the average value of the expression of genes. Rows represent genes with significant changes (by means of Kruskal -Wallis test). Columns represent age groups: 20–29, 30–39. 40–49, 50–59, 60–69, 70–79 years. Lower values are represented in red while green means higher values. In the top right corner is the related protein interaction network extracted from the STRING database.

Finally, as depicted in Fig. 16, we found a significant (*p* value $\le$ 0.01 Kruskal Wallis test) variation in the following genes with respect to tissues: CHI3L1 in the *kidney*, B2M in the *lung*, CNTF in *muscle*, AR in *nerve tibial*, CX3CL1 in the *pancreas*, TSC1 in the *uterus*, and NGF in *Blood*.

The patterns for all the genes obtained by using the proposed framework have been reported as boxplots and are available at https://github.com/hguzzi/DiabetesAging/tree/main/boxplot.

**Figure 17:**
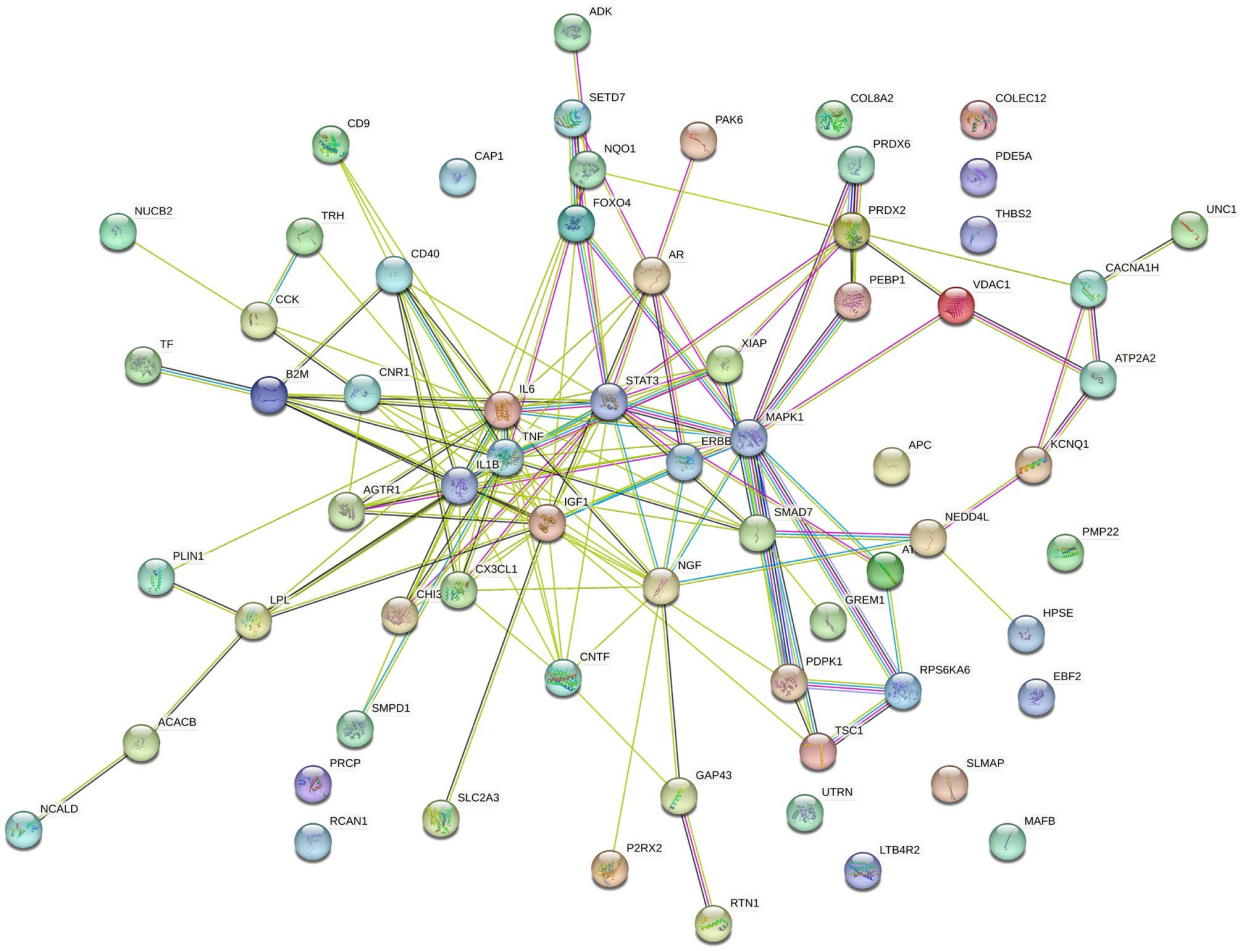
Figure depicts the protein interaction network induced by all the genes with a different average expression depending on age groups. The network has 66 nodes and 147 edges. The average node degree is 4.45 and has a *p*-value < 1.0e-16 to be generated at random. This means that the proteins have more interactions themselves than what would be expected for a random set, indicating that as a group the proteins are at least partially connected biologically.

**Figure 18:**
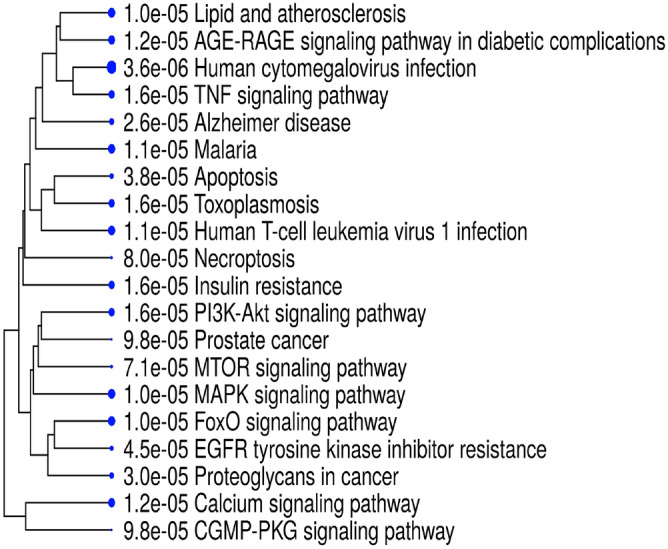
Figure depicts the functional enrichment of the genes with a different average expression depending on age group. The dendrogram shows some interesting functions such as Insulin Resistance, AGE–RAGE pathway, Lipid and Atherosclerosis.

To complete our study we built the network of interactions of all the significant genes found by using the STRING database. The resulting network has 65 interacting genes and 147 interactions as reported in Fig. 17. Functional enrichment of this network has been evaluated and results are reported in Fig. 18, where the dendrogram shows some interesting detected functions.

**Figure 19:**
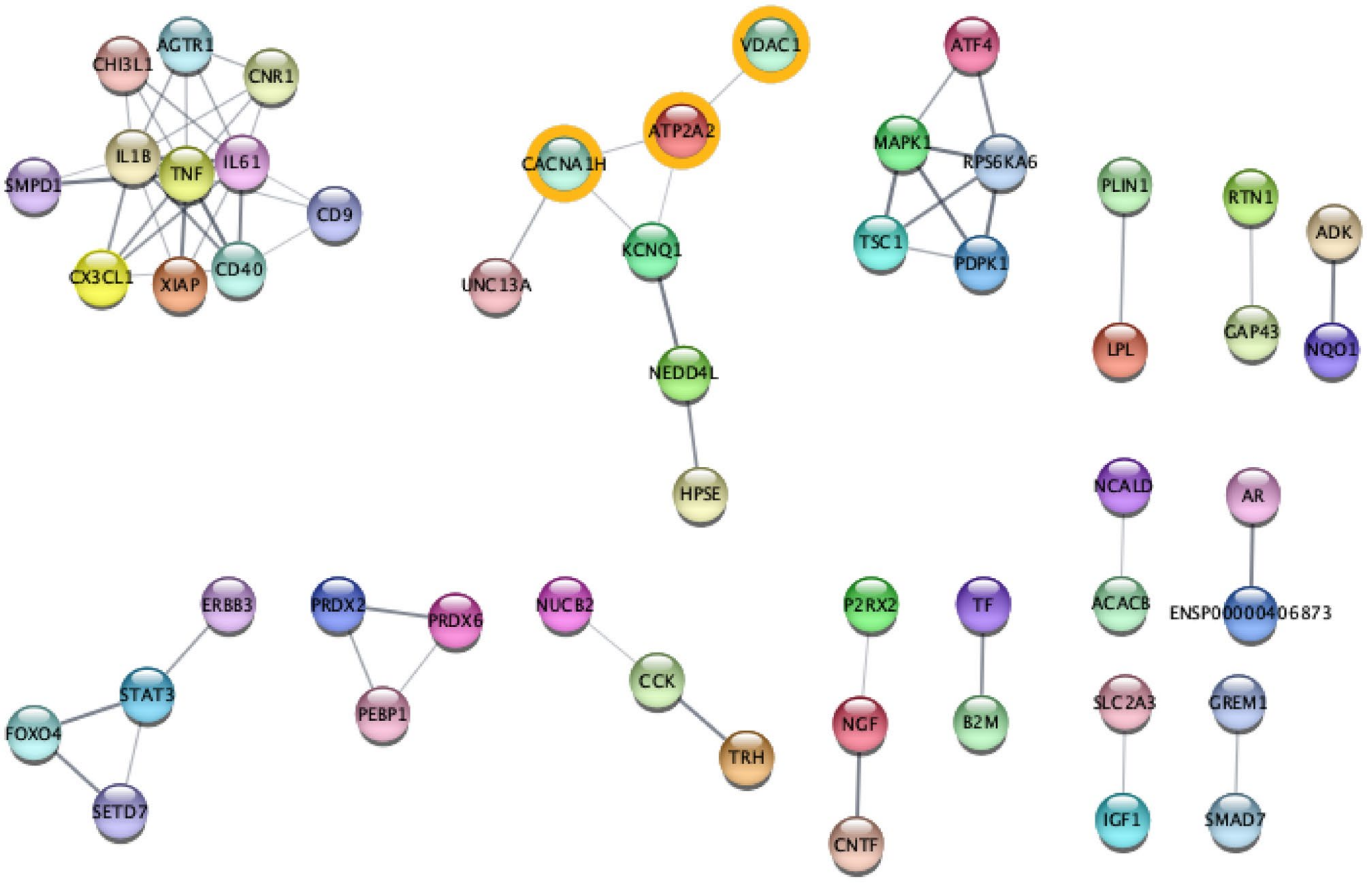
Clustered Network of all the interactors using Markov Clustering MCL (inflation parameter set to 3.0). The analysis reveals the emergence of seven subnetworks, each corresponding to a synergistic relation of the genes.

We further analysed the network by means of cluster analysis made with Markov Clustering Software^47^, which reveals the insurgence of four subnetworks as reported in Fig. 19.

**Figure 20:**
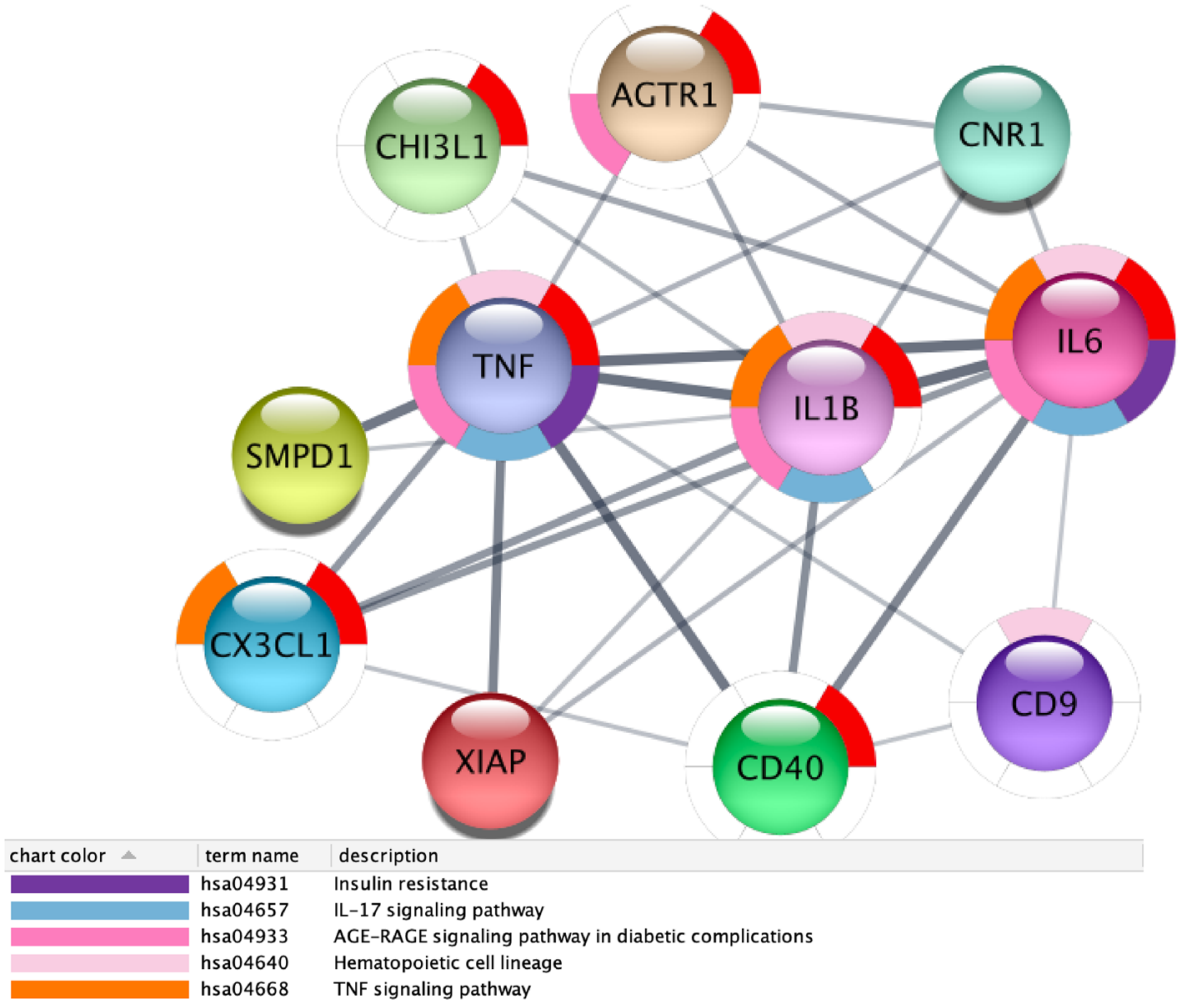
Figure depicts the protein interaction network induced by all the genes having a different average expression depending on age group. The table shows the main enriched function. Functional Enrichment shows that the subnetwork is associated with pathways related to diabetes: Insulin resistance, AGE–RAGE2 pathway, and Tumor Necrosis alpha.

First subnetwork shows a set of genes (i.e., CHI3L1, AGTR1, SMPD1, CX3CL1, XIAP, CD40, CD9, IL6, and CNR1) centred around TNF and IL1B as depicted in Fig. 20.

**Figure 21:**
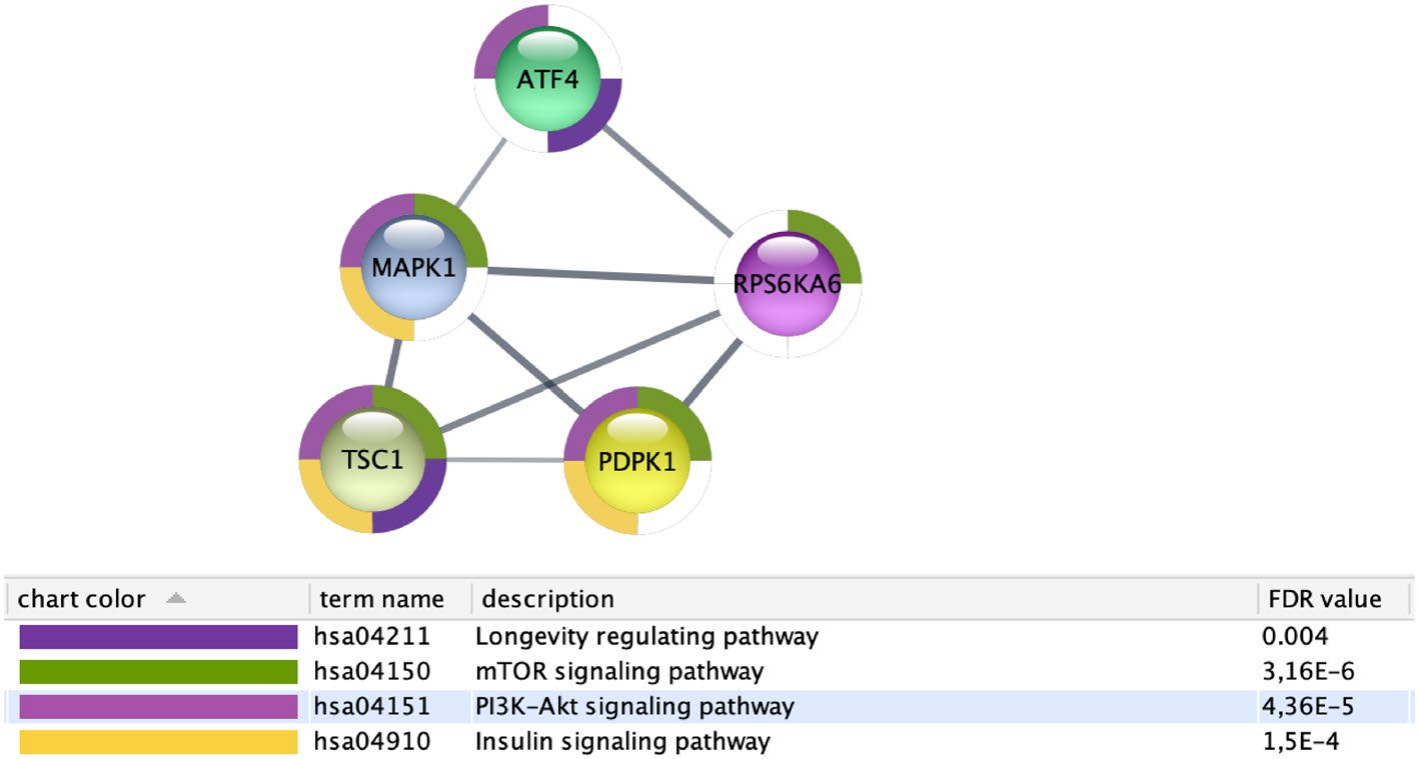
Figure depicts the protein interaction network induced by all the genes having a different average expression depending on age group. Table shows main enriched function. The following proteins and the related pathways are involved in diabetes and ageing: longevity regulating pathway (MAPK1), Insulin Signaling Pathway (PDPK1), and mTOR.

This network is related to the following KEGG pathways (FDR less than 0.01): Insulin resistance, TNF1 and IL1B pathways, hematopoietic cell lineage and AGE–RAGE. The subnetwork, depicted in Fig. 21, shows a set of proteins centred around MAPK gene responsible for (i) the mTOR signalling pathway, (ii) the PT3K-Akt pathway and (iii) the insulin signalling pathway. The network confirms that many mechanisms related to diabetes mellitus are deregulated with age.

**Figure 22:**
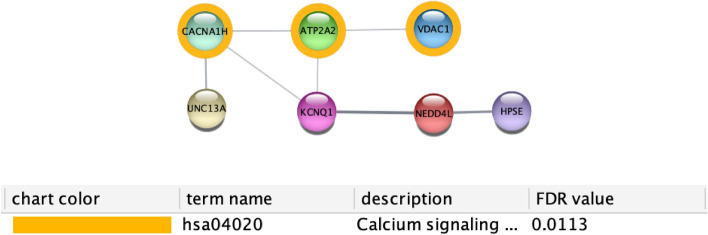
Figure depicts the protein interaction network induced by all the genes having a different average expression depending on age groups. Table shows the main enriched function.

The network depicted in Fig. 22, highlights the changes in proteins related to calcium signalling. Finally, the 54 obtained networks are available at https://github.com/hguzzi/DiabetesAging/tree/main/networkanalysis.

## Discussion

Elderly patients diagnosed with T2DM usually have more than two comorbidities^48^. T2DM comorbidities span a wide class such as (i) cognitive and visual impairment, (ii) incontinence, (iii) hearing loss, (iv) severe hyper/hypoglycemic episodes and adverse cardiovascular events, and (v) peripheral and autonomic neuropathy. Patients presenting pathologies such as obesity, geriatric syndromes, or cardiovascular risk factors, may also have an increased mortality risk related to diabetes mellitus disease^49^.

Assuming that the increased risk of comorbidity in elderly could be related to changes in the basal expression of genes, we selected all the genes related to comorbidities and found that a number of them present significant changes with age, thus confirming our hypothesis. We analysed such genes and found that they are significantly enriched in some aspects related to diabetes mellitus, as depicted in Fig. 18 where functional enrichment of the genes with a different expression across different age groups is reported.

Results show a relatively high number of genes related to comorbidities whose basal expression modifies with age. The analysis of such genes in different brain tissues shows the deregulation of many genes and pathways (see Figs. 6, 7, 8, 9, 10, 11).

These changes may explain the particular insurgence of *neurological* T2DM comorbidities in the elderly. The network among these genes is reported in Fig. 17 where it can be noted some modulated pathways potentially responsible for brain damage were identified. Moreover, by analyzing the obtained networks, we found the VEGFA-VEGFR2 pathway^50^ whose modifications have been demonstrated to correlate with a metabolic syndrome^51^.

Functional enrichment also indicates a change in the pathway related to insulin signalling and the metabolism of regulation of glucose and lipid^52,53^. In the adipose tissue, as shown in Fig. 13, we found significant changes in glucose-related pathways as in^54^, where research topics regarding adipose tissue and its dysfunction and changes in gene expression with age are reported. As reported in^55^, there are beneficial effects of cardiac microvascular protection in diabetes mellitus through PRDX2 pathways. We found that the downregulation of the PRDX2 gene in elderly patients implies an added risk of cardiac disease in this age group. In parallel, we found some genes related to the AGE-RAGE signalling pathway as in^56^. The dysregulation of the AGE-RAGE pathway results in Advanced glycation end products triggering oxidative stress. Consequently, diabetes mellitus in older patients amplifies the modification of this pathway, thus enhancing the triggering of oxidative stress and inflammation.

Since obesity entails an accelerated ageing process, thus inducing an inflammation state, we find many genes responsible for comorbidities are expressed in the adipose tissue. We may thus state that reducing obesity induces the reduction of ageing process and inflammation, pointing the way towards new therapies.

We found two genes, the CCK, and the KCNQ1, that are expressed in the aorta tissue, whose expression is significantly modified with age. Both are related to pancreatic secretion pathways^57^. KCNQ1 is also associated with diabetic nephropathy (DN) that can be considered the primary cause of end-stage renal disease (ESRD)^58^. Also, we identified IL1B and TNF genes that change with age in liver tissue as in Fig. 14. The role of IL1B in diabetes mellitus has been investigated recently in^59^.^60^ excludes a prominent role of IL1B in T2DM, while we found that there is evidence of a function of IL1B in the regulation of postprandial glucose^60^, and in glucose homeostasis^61^. Our findings suggest that dysregulation of IL1B levels may play a physiological role in IL1B and insulin regulation, exacerbating postprandial inflammation. The synergistic dysregulation of IL1B and TNF in elderly patients may suggest the tailoring of therapeutic strategies for the elderly^62^. We found deregulation of Beta 2 microglobulin (B2M) in the lung as depicted in the heatmap of Fig. 16. This implies the deregulation of cytokine regulation and PI3-AKT pathways. The literature notes that B2M has a role in oxidative stress in older patients^63^. The dysregulation of B2M may have an impact on the regulation of cellular functions. It also has a documented role in kidney dysfunctions in diabetes mellitus patients. Here we provide that B2M may have a supplementary role in lung inflammation^64,65^.

In this work, we used only in-silico data, and we analysed the change of the basal expression of such genes in the healthy population. This may be a limitation of this work regarding causality relation. Nevertheless, we think that screening all the possible candidates and selecting a limited group of candidates and defining relations among ages and comorbidities may help in - for instance - studying new strategies for preventing and treating comorbidities occurrence.

The specificity of the findings for diabetes mellitus relies on the initial selection of candidate genes in the T2DiACoD database that stores only genes known to be related to T2DM comorbidities. All the genes contained in the T2DiACoD database have a direct relation with T2DM comorbidities. The added value of our manuscript is the analysis of how these genes also present a modulation with age. The age-dependent modulation can be an additional risk factor for developing diabetes comorbidities. The analysis of the causal relationship may constitute a follow-up of this work.

## Methods

To study genes and their changes with respect to ageing tissues, we queried available databases using the proposed framework whose architecture is reported in Fig. 2. This framework uses (i) the T2DiACoD database for selecting candidate genes, (ii) the GTEx portal for gene expression at the tissue level, and (iii) the STRING database to build interaction networks. We briefly describe the aforementioned data sources, as well as the bioinformatics pipeline reported at the bottom part of Fig. 2.

### Data sources

The proposed framework is based on the following data sources: T2DiACoD database^39^, GTEx database^40^, and STRING network database^66^. T2DiACoD contains genes and non-coding transcripts related to complications associated with type 2 diabetes mellitus. It is the result of research into T2DM which links genes to disease. In particular, the authors focused on comorbidities and complication diseases such as atherosclerosis, nephropathy,*diabetic retinopathy, diabetic neuropathy*, and cardiovascular diseases. The database, populated by mining the literature and other data sources, stores 650 genes and 34 microRNAs related to comorbidities. *Genes are associated with the comorbidities as reported in the following Table* 2. *The number of samples for each tissue is variable as reported in Table*3

**Table 2:** Number of Genes for each disease as stored in the T2DiACoD database.

T2 associated disease	Number of genes
Atherosclerosis	115
Cardiovascular	172
Diabetic nephropathy	403
Diabetic neuropathy	130
Diabetic retinopathy	161

**Table 3:** Number of samples for each tissue.

Tissue	Samples
Adipose subcutaneo	663
Adipose visceral	541
Adrenal gland	258
Artery aorta	864
Brain anterior	176
Brain caudate	246
Brain cerebellar	215
Brain cortex	255
Brain frontal cortex	209
Brain hippocampus	197
Brain hypotalamus	202
Brain nucleus accumbengs	246
Brain putamen Basal ganglia	205
Brain substantia nigra	139
Breast	459
Colon	373
Kidney	85
Liver	226
Lung	578
Muscle skeletal	803
Nerve tibial	619
Pancreas	328
Uterus	142
Whole blood	755

The GTEx data portal represents one of the most commonly used sources for collecting whole-genome sequencing and RNA-seq data in individuals. For each sample, GTEx provides patient information, such as tissue of provenance, sex and age (grouped into six classes). The current version of the GTEx database (v8 accessed on September 25th stores 17382 samples of 54 tissues of 948 donors, see at https://gtexportal.org/home/tissueSummaryPage) is available on the web and offers an easy to use query interface and visualisation of data in tissues used in many ageing-related studies^3,67–69^.

The STRING protein interaction network database is used to build protein networks. It collects evidence of interactions from many sources, from text-mining and computational prediction to annotated experiments. For each interaction, it provides the source of the association (e.g. physical or computational) and a reliability score in the 0..1 interval. *We considered only physical interactions with a reliability value higher than 0.400.*

### Data analysis

The above reported data sources have been used by framework proposed herein to perform the following steps. The framework allows the user to query the T2DiACoD database to retrieve genes related to T2DM comorbidities contained in the database. Each gene is then used to query the GTEx data portal in order to retrieve all the available expressions. Results obtained from the GTEx portal are also associated with tissues and age information. Samples are grouped using tissues and each tissue expression is analysed. Also, median values of gene expression is calculated with respect to age classes: 20–29, 30–39, 40–49, 50–59, 60–69, 70–79 years. We then selected those genes whose average values of the expression are monotonically increasing or decreasing in that age interval. For each gene we calculated the significance of the difference in the expression between the intervals by means of Kruskal–Wallis test. A *p*-value less than 0.01 (after correction for multiple tests) was considered significant. *Since we verified that normality distribution does not hold for some classes, we decided to apply a Kruskal Wallis test with respect to the ANOVA. The calculated* *p*-value *and testing correction has been performed by using the Bonferroni correction method. *

From the STRING database^70^ we extracted the protein interaction networks induced by increasing or decreasing genes. For each network we computed the functional enrichment by means of the STRING web server focusing on gene ontology (GO) Biological Process and KEGG database pathways enriched with FDR *p*-value less than 0.05.

### Data and code availability

Network visualisation was performed through a Cytoscape app (http://apps.cytoscape.org/apps/stringapp). The analysis was implemented in the R programming Language. Data and Code are available at https://github.com/hguzzi/DiabetesAging. More data are available upon reasonable request. Heatmaps used in this article have been generated by using the on line software http://www.heatmapper.ca/expression/^71^.

## Conclusion

We proposed a framework that by focusing on type 2 diabetes (T2DM) comorbidities, can gather data from gene databases. By measuring gene variations over time, we obtained significant results as regards the gene responsible for comorbidities. First we selected from the T2DiACoD database the list of genes related to comorbidities. We then extracted networks of proteins connecting them and, at the same time, analysed their pattern considering age as factors. We *proved* the action of certain genes that could be used to develop specific therapies. As the proportion of elderly people grows the number of cases of T2DM increases. This research could help in understanding the hidden link between age and diabetes, thereby fostering the development of new strategies to prevent the effects of ageing and also improve the treatment or prevention of Type 2 diabetes mellitus.

## Data Availability

Website https://github.com/hguzzi/DiabetesAging/tree/main/boxplot contains all the figures, data and networks used in this work.
